# Washington’s State Dinners

**Published:** 1881-05

**Authors:** 


### WASHINGTON'S STATE DINNERS.

DURING General Washington's administration, a State dinner was a solemn affair, as the guests acted as if dining off "funeral bak'd meats." Mr. Maclay, one of the first two Senators from Pennsylvania, gives us, through an entry in his diary, a glimpse of one of these formal dinners:

THURSDAY, Aug. 27, 1789.—A little after four o'clock I called on Mr. Bassett, of Delaware State, and we went to the President's for dinner. The company were President and Mrs. Washington, Vice-President and Mrs. Adams, the Governor (Mifflin) and his wife, Mr. Jay and wife, Mr. Langdon and wife, Mr. Dalton and a lady—perhaps his wife—Bassett, myself, Lee, Lewis, and the President's two secretaries. The President and Mrs. Washington sat opposite each other in the middle of the table. The two secretaries one at each end.

It was a great dinner, and the best of the kind I ever was at. The room, however, was disagreeably warm.

First were soup, fish, roasted and baked meats, gammon, fowl, etc. This was the dinner. The middle of the table was garnished in the usual tasty way, with small images, artificial flowers, etc. The dessert was fruit, apple pies, puddings, etc.; then ice-cream, jelly, etc.; then watermelons, muskmelons, apples, peaches and nuts.

It was the most solemn dinner I was ever at. Not a health drank, scarce a word said until the cloth was taken away. Then the President, filling a glass with great formality, drank the health of every individual around the table. Everybody imitated him, changed glasses, and such a buzz of "Health, sir," "Health, madam," "Thank you, sir," and "Thank you, madam," I had never heard before.

The ladies sat a good while, and the bottles passed about, but there was a dead silence almost. Mrs. Washington at last withdrew with the ladies. I expected the men would now begin, but the same silence remained. The President told of a New England clergyman who had lost his hat and wig in passing a river called the Bronx, and he smiled and everybody else laughed. He now and then said a sentence or two on some common subject, and what he said was not amiss. Mr. Jay tried to make a laugh by mentioning the caricature of the Duchess of Devonshire assisting in carrying Fox's election.

The President kept a fork in his hand when the cloth was taken away, I thought for the purpose of picking nuts ; he ate none, but played with the fork, striking on the edge of the table.

We did not sit long after the ladies retired. The President rose and went upstairs to drink coffee. The company followed. I took my hat and went home.

